# Using Ultrasound-Based Multilayer Perceptron to Differentiate Early Breast Mucinous Cancer and its Subtypes From Fibroadenoma

**DOI:** 10.3389/fonc.2021.724656

**Published:** 2021-12-01

**Authors:** Ting Liang, Junhui Shen, Shumei Zhang, Shuzhen Cong, Juanjuan Liu, Shufang Pei, Shiyao Shang, Chunwang Huang

**Affiliations:** ^1^ Department of Ultrasound, Guangdong Provincial People’s Hospital, Guangdong Academy of Medical Sciences, Guangzhou, China; ^2^ Department of Ultrasound, Affiliated Hospital of Guangdong Medical University, Zhanjiang, China; ^3^ Department of Rehabilitation Medicine, Guangdong Provincial People’s Hospital, Guangdong Academy of Medical Sciences, Guangzhou, China; ^4^ Department of Ultrasound, Guangzhou Eighth People’s Hospital, Guangzhou Medical University, Guangzhou, China

**Keywords:** ultrasound, mucinous breast carcinoma, fibroadenoma, multilayer perceptron, machine learning

## Abstract

**Objectives:**

Mucinous breast cancer (MBC), particularly pure MBC (pMBC), often tend to be confused with fibroadenoma (FA) due to their similar images and firm masses, so some MBC cases are misdiagnosed to be FA, which may cause poor prognosis. We analyzed the ultrasonic features and aimed to identify the ability of multilayer perceptron (MLP) to classify early MBC and its subtypes and FA.

**Materials and Methods:**

The study consisted of 193 patients diagnosed with pMBC, mMBC, or FA. The area under curve (AUC) was calculated to assess the effectiveness of age and 10 ultrasound features in differentiating MBC from FA. We used the pairwise comparison to examine the differences among MBC subtypes (pure and mixed types) and FA. We utilized the MLP to differentiate MBC and its subtypes from FA.

**Results:**

The nine features with AUCs over 0.5 were as follows: age, echo pattern, shape, orientation, margin, echo rim, vascularity distribution, vascularity grade, and tumor size. In subtype analysis, the significant differences were obtained in 10 variables (p-value range, 0.000–0.037) among pMBC, mMBC, and FA, except posterior feature. Through MLP, the AUCs of predicting MBC and FA were both 0.919; the AUCs of predicting pMBC, mMBC, and FA were 0.875, 0.767, and 0.927, respectively.

**Conclusion:**

Our study found that the MLP models based on ultrasonic characteristics and age can well distinguish MBC and its subtypes from FA. It may provide a critical insight into MBC preoperative clinical management.

## Introduction

Mucinous breast cancer (MBC) accounts for about 2% of all invasive breast carcinomas (1), whose prevalence is reported to be 1%–6% of all breast cancers (2). According to WHO classification, MBCs are classified as pure (pMBCs) and mixed MBCs (mMBCs) based on the lesions’ mucin production. The pMBC consists exclusively of tumor tissue with a mucinous component above 90%, while mMBC with mucinous areas covers more than 50% but <90% of the total area and admixes usually with an infiltrating ductal epithelial component (2, 3). For MBC, metastatic disease rate ranges were reported from 12% to 14% in the case series (4). pMBC has a better overall survival than mMBC (3). Clinically, MBCs are palpable and firm masses and often tend to be confused with fibroadenomas (FAs). Some of them were misdiagnosed as FAs, delaying treatment, resulting in axillary node metastasis, chemotherapy, and shortened disease-free survival. Thus, it is essential to precisely differentiate early MBCs and their subtypes from FAs through radiological methods.

Mammography, magnetic resonance imaging (MRI), and ultrasound (US) are the main imaging techniques for discovering breast masses and preliminarily judging their histological properties. The efficiency of mammographic mass detection is low in dense breast tissues and in MBCs (5, 6). MRI is very expensive and has been associated with high false-positive rate for breast cancers (7). In contrast, US is inexpensive, non-radioactive, and widely available, and is therefore the preferred radiological means for diagnosing breast masses, especially in dense breast tissues (8).

Currently, the American College of Radiology Breast Imaging Reporting and Data System’s (ACR BI-RADS) lexicon is the most commonly implemented evaluating system for breast lesions. In practice, some MBCs and FAs have the similar images. Based on the lexicon, some MRI studies focused on differentiating MBCs and FAs (9, 10). Despite the fact that one of such studies has selected optimal characteristics related with MBCs, it has not analyzed the association with the subtypes (10). Regrettably, previous US studies have just presented the features of each MBC subtype (11–13). They failed to predict MBCs, subtypes, and FAs based on a single clinical or ultrasonic feature. Therefore, we should conduct the integrated approach, such as machine learnings.

As one of machine learnings, multilayer perceptron (MLP) performs very well on nonlinear data (14), has high fault tolerance, and can solve complex problems (15, 16). Previous ultrasonic studies have performed the classification well for malignant tumors using MLP (17, 18). To our best knowledge, there is no ultrasonic study that analyzes the ultrasonic characteristics to distinguish MBC and its subtypes from FA using MLP. In this study, we analyzed the ultrasonic features of MBC subtypes and FA using MLP and identified whether MLP can perform the classification well to improve the diagnostic performance for early MBC subtypes and FA.

## Materials and Methods

### Participants and Study Design

Ethical approval was approved by Research Ethics Committee of Guangdong Provincial People’s Hospital for this retrospective study, and the informed consent requirement was waived due to the retrospective study. The histological characteristics of the included breast masses were gathered from pathology reports. From January 1, 2013 to December 30, 2019, 61 pMBCs and 31 mMBCs patients were enrolled in this retrospective study. Then, from January 1, 2019 to May 31, 2019, 101 consecutive FAs were enrolled in this retrospective study because FAs were the most common. All patients’ age range was 15–82 years old, and mean age was 43.64 ± 14.40 years old.

The inclusion criteria were the following: (1) breast masses identified as pMBCs, mMBCs, or FAs through histological examination; (2) patients with single mass; and (3) patients of MBC without axillary node and distant metastasis.

The exclusion criteria were the following: (1) lesions that were metastatic tumors; (2) patients exposed to systemic hormone therapy or adjuvant chemotherapy; (3) lesions larger than 6 cm.

### Ultrasonic Image Acquisition and Interpretation

Ultrasonic image acquisition was captured using a 14-MHz linear transducer (Toshiba Aplio 500, Canon Medical Systems Corp., Tokyo, Japan). Images of the masses were collected in a standard manner, containing at least two orthogonal planes (the radial and antiradial planes or transverse and longitudinal planes), by two breast radiologists (reader 1 with 10 and reader 2 with 5 years’ experience, respectively) following the ACR BI-RADS fifth edition classification scheme. As directed by the guide and previous article (19), the two radiologists kept a strict record of US features. Both were blind to the histological outcome but not to ages. The ultrasonic characteristics comprised of 10 items: nodulous echo pattern, shape, orientation, margin, posterior features, tumor size, calcifications, echogenic rim, vascularity distribution, and vascularity grade. Detailed feature descriptions are presented in the data supplement (**Appendix 1**).

For the records of each ultrasonic feature, any disagreements between the two readers were resolved by final consensus following discussion.

### Statistical Analysis

Statistical analysis was conducted with the SPSS software (Version 22.0, IBM Corp., Armonk, NY, USA). The statistical significance levels were two-sided, and *p* < 0.05 was deemed to be statistically significant.

### Comparison of the MBC and FA Groups and Multiple Comparisons of pMBC, mMBC, and FA

Depending on ultrasonic features and age, the differences between MBC and FA were evaluated. Continuous variables were compared using the Mann–Whitney U test or t-test. Categorical variables were compared using the chi-square test or Fisher’s exact test.

With respect to ultrasonic features and age, the multiple comparisons among pMBC, mMBC, and FA were assessed. Hereby, continuous variables were compared using the least significance difference (LSD), whereas categorical variables were compared using the Kruskal–Wallis test.

### Predicting MBC and FA

For all ultrasonic features and age, the receiver operating characteristic curves (ROCs) were plotted using ROC in SPSS Statistics. According to the curves, the respective area under curves (AUCs), sensitivity, and specificity were calculated and given automatic in SPSS Statistics. Youden index is equal to sensitivity plus specificity minus one. The sensitivity, specificity, and Youden index of those features, whose AUCs were over 0.5, were presented.

In addition, for distinguishing MBC from FA, the Multilayer Perceptron in SPSS Statistics was used to complete MLP analysis. After completing the process, the AUC of MLP and the importance of features were given automatic in SPSS Statistics.

### Predicting MBC Subtypes and FA

MLP was used to distinguish MBC subtypes from FA, and the corresponding methods are shown in the previous paragraph. The AUC of MLP and the importance of features were provided.

### Clinical Use

The two models of MLP can be saved in the XML file. When there are new data, you can directly call this file in the SPSS software to calculate the probability of the type of MBC or FA in the data supplement (**Appendix 2**).

## Results

### Comparison of MBC and FA and Multiple Comparisons of pMBC, mMBC, and FA

Patients’ ages and 10 detailed ultrasonic characteristics are revealed in **Table 1**. The prevalence of FA, MBC, pMBC, and mMBC were 52% (101/193), 48% (92/193), 32% (61/193), and 16% (31/193), respectively.

**Table 1:** Patients’ age and ultrasonic characteristics in FA, MBC, and subtypes.

	MBC				*P_1_*	*P_2_*	*P_3_*	*P_4_*
	pMBC	mMBC	(pMBC + mMBC)	FA				
	(n = 61)	(n = 31)	(n = 92)	(n = 101)				
**Age (year)**					0.000	0.000	0.143	0.000
Mean ± SD	52.85 ± 13.02	48.87 ± 13.51	51.51 ± 13.25	36.47 ± 11.38				
**Echo pattern**					0.036	0.334	0.000	0.000
Hyperechoic	0 (0)	0 (0)	0 (0)	1 (1%)				
Complex cystic and solid	1 (2%)	1 (3%)	2 (2)	0 (0)				
Hypoechoic	30 (49%)	19 (61%)	49 (53%)	84 (83%)				
Isoechoic	28 (46%)	6 (19%)	34 (37%)	12 (12%)				
Heterogeneous	2 (3%)	5 (16%)	7 (8%)	4 (4%)				
**Shape**					0.000	0.315	0.001	0.000
Oval	7 (11%)	1 (3%)	8 (9%)	36 (36%)				
Round	3 (5%)	1 (3%)	4 (4%)	3 (3%)				
Irregular	51 (84%)	29 (94%)	80 (87%)	62 (61%)				
**Margin**					0.000	0.294	0.001	0.000
Circumstance	8 (13%)	1 (3%)	9 (10%)	36 (36%)				
Not circumstance	53 (87%)	30 (97%)	83 (90%)	65 (64%)				
**Orientation**					0.057	0.885	0.027	0.010
Parallel	46 (76%)	23 (74%)	69 (75%)	88 (87%)				
Not parallel	15 (24%)	8 (26%)	23 (25%)	13 (13%)				
**Posterior feature**					/	/	/	0.561
No posterior feature	30 (49%)	18 (58%)	48 (52%)	54 (53%)				
Enhancement sound	28 (46%)	12 (39%)	40 (43%)	31 (31%)				
Shadowing	3 (5%)	1 (3%)	4 (4%)	14 (14%)				
Combined pattern	0 (0)	0 (0)	0 (0)	2 (2%)				
**Calcification**					0.000	0.065	0.030	0.001
In a mass	22 (36%)	16 (52%)	38 (41%)	20 (20%)				
None	39 (64%)	15 (48%)	54 (59%)	81 (80%)				
**Echogenic rim**					0.351	0.129	0.001	0.004
None	47 (77%)	28 (90%)	75 (82%)	97 (96%)				
Enhanced	14 (23%)	3 (10%)	17 (18%)	4 (4%)				
**Vascularity distribution**					0.028	0.847	0.012	0.003
Absent	24 (39%)	12 (39%)	36 (39%)	62 (61%)				
Vessels in rim	2 (3%)	2 (6%)	4 (4%)	34 (34%)				
Internal	35 (57%)	17 (55%)	52 (57%)	4 (4%)				
**Vascularity grade**					0.011	0.455	0.028	0.004
Grade I	23 (38%)	11 (35%)	34 (37%)	62 (61%)				
Grade II	16 (26%)	6 (19%)	22 (24%)	13 (13%)				
Grade III	17 (29%)	8 (26%)	35 (38%)	19 (19%)				
Grade IV	5 (8%)	6 (19%)	11 (12%)	7 (7%)				
**Size (cm)**					0.000	0.007	0.280	0.000
Mean ± SD	2.71 ± 1.31	2.47 ± 1.06	2.63 ± 1.23	1.91 ± 0.74				

P_1_, pMBC vs. FA; P_2_, mMBC vs. FA; P_3_, pMBC vs. mMBC; P_4_, MBC vs. FA; pMBC, pure mucinous breast cancer; mMBC, mixed mucinous breast cancer; MBC, mucinous breast cancer; FA, fibroadenoma.

There were significant differences in 10 variables (*p*-value range, 0.000–0.004) between MBC and FA, except posterior feature **(** **Table 1** **)**.

In subtype analysis, one-way ANOVA analysis found that there were statistically significant differences in 10 variables (p-value range, 0.000–0.037) between pMBC, mMBC, and FA groups as a whole, except posterior feature (p-value, 0.630). Furthermore, the multiple comparisons of the 10 variables with statistically significant differences are outlined in **Table 1**.

### Predicting MBC and FA

The AUCs of all the 11 variables for MBC and FA were calculated. The nine AUCs over 0.5 were as follows: age, echo pattern, shape, orientation, margin, echo rim, vascularity distribution, vascularity grade, and size. Their corresponding AUCs and sensitivity, specificity, and Youden index of the above predictors for differentiating MBC from FA are displayed in **Table 2**. The AUCs of posterior feature and calcification were below 0.5, indicating that these two variables could not distinguish between MBC and FA.

**Table 2:** Sensitivity, specificity, and Youden index of nine features for differentiating MBC from FA.

	Sensitivity	Specificity	Youden Index	AUC
Age	76.10%	81.20%	0.57%	0.817
Echo pattern	44.60%	84.20%	0.288	0.635
Shape	87%	38.60%	0.256	0.634
Orientation	25%	81.10%	0.061	0.571
Margin	90.20%	36.60%	0.268	0.648
Echogenic rim	16.30%	96%	0.123	0.571
Vascularity Distribution	60.90%	61.40%	0.223	0.608
Vascularity grade	63%	60.40%	0.234	0.611
Size	70.70%	63.40%	0.341	0.683

The AUCs (area under curve) of the nine features were over 0.5.

**Figure 1:**
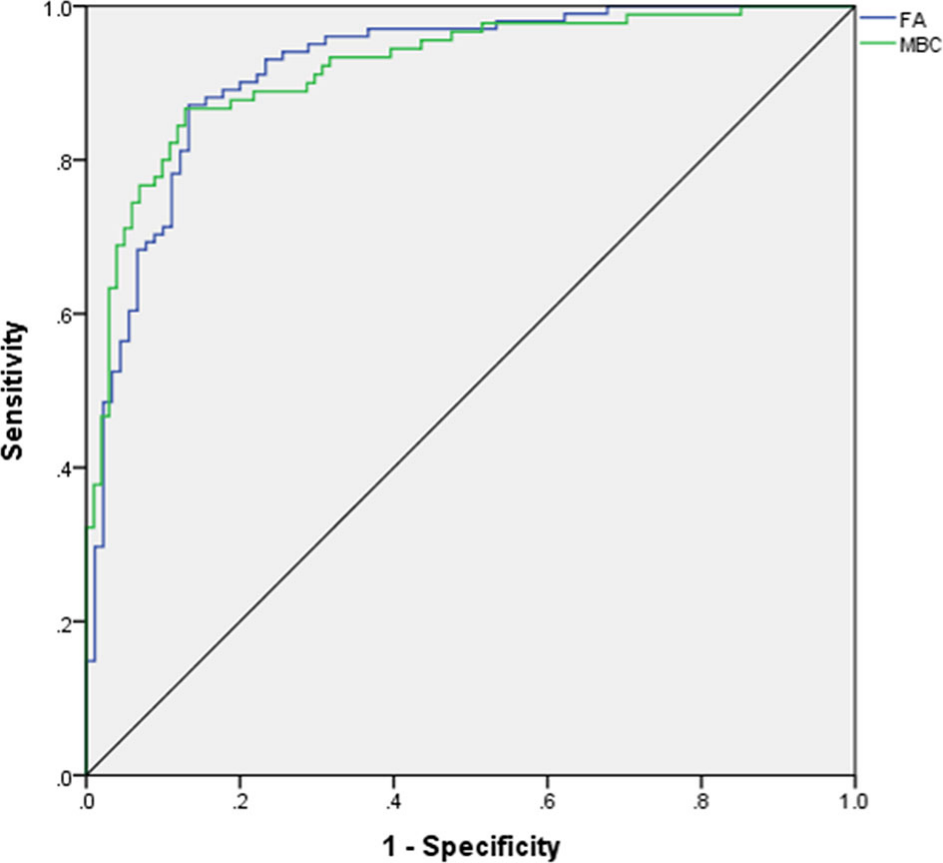
ROC for differentiating mucinous breast carcinoma from fibroadenoma. ROC, receiver operating characteristic curve; AUC, area under curve; FA, fibroadenoma; MBC, mucinous breast carcinoma.

**Figure 2:**
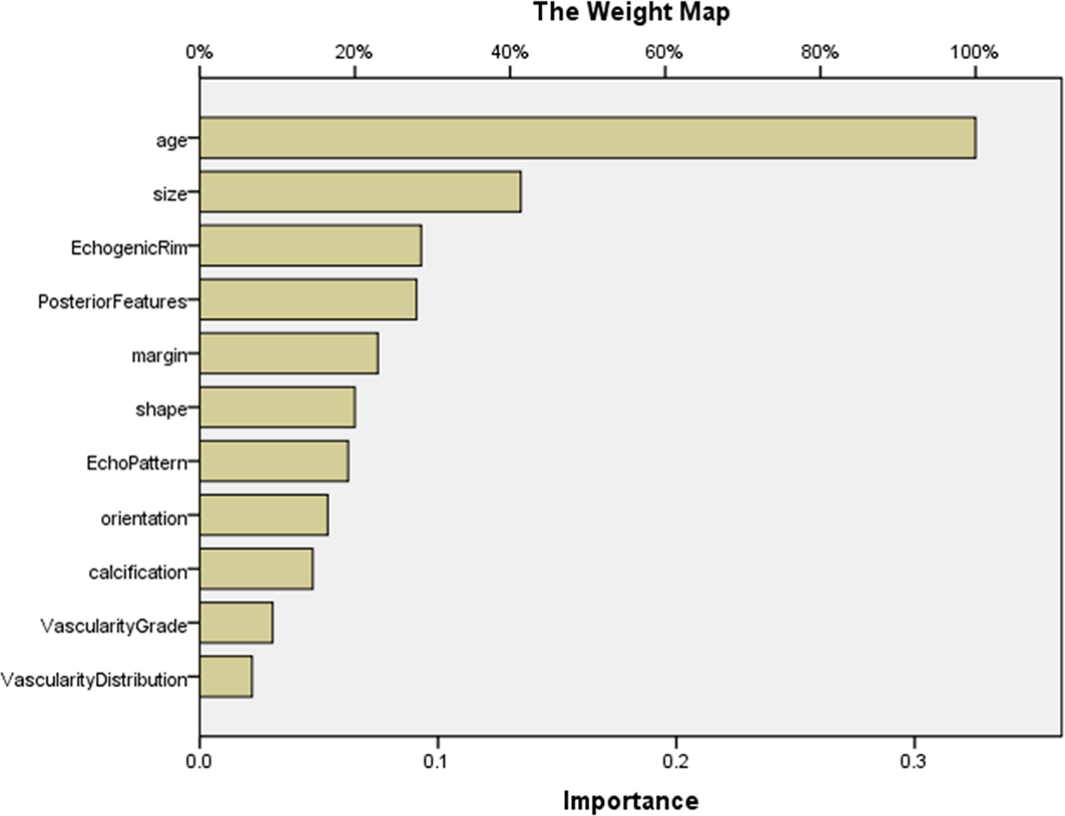
The importance of features in MLP for predicting MBC and FA. The map could present the importance of each feature. The longer the bar represented by this feature, the greater its weight. According to the map, the top 5 features were age, size, echogenic rim, posterior features, and margin. MLP, multilayer perceptron.

For predicting MBC and FA, the AUCs of MLP were calculated, and the ROCs of MLP are plotted in **Figure 1**. According to ROCs, AUCs were both 0.919. The importance of the features is depicted in **Figure 2**.

### Predicting MBC Subtypes and FA

**Figure 3:**
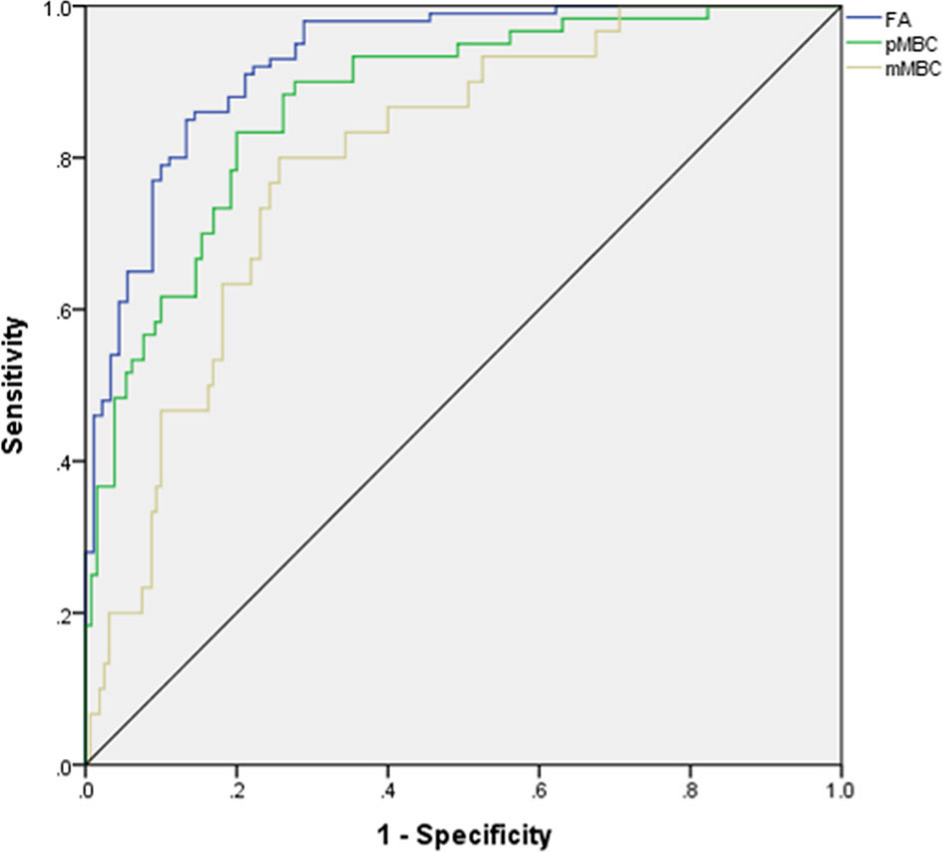
ROC for differentiating mucinous breast carcinoma or subtypes from fibroadenoma. ROC, receiver operating characteristic curve; AUC, area under curve; FA, fibroadenoma; MBC, mucinous breast carcinoma; pMBC, pure mucinous breast carcinoma; mMBC, mixed mucinous breast carcinoma.

**Figure 4:**
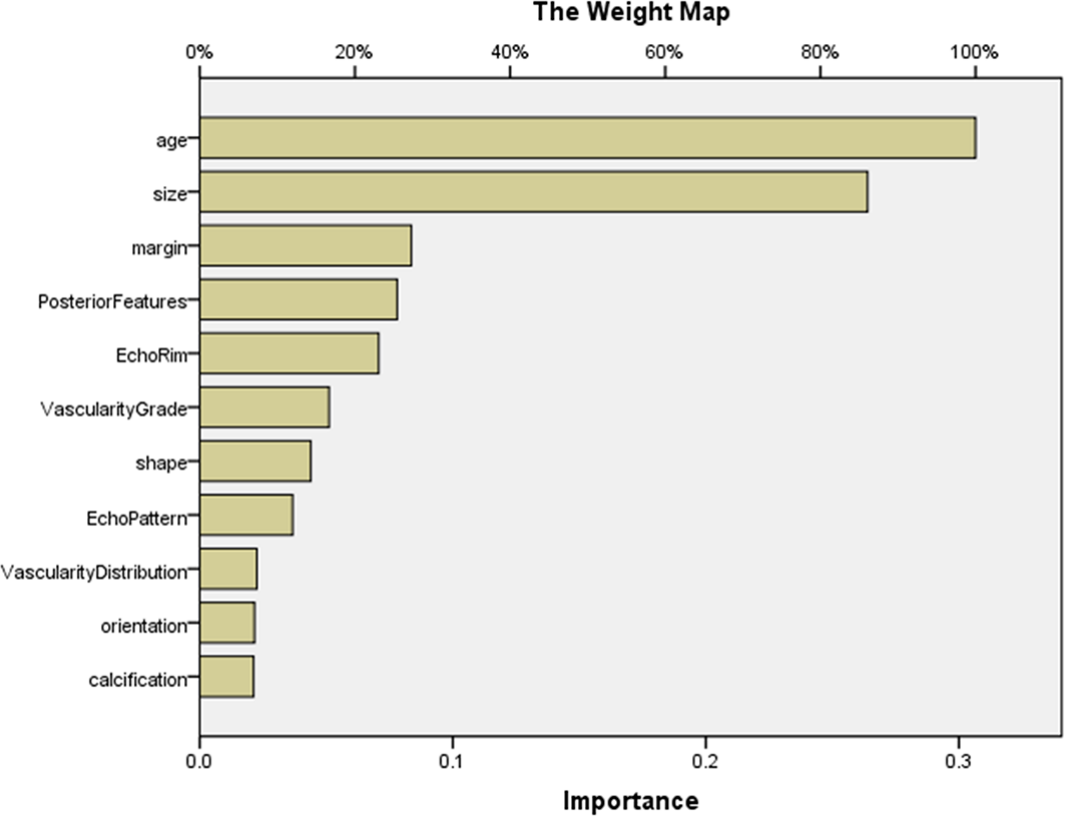
The importance of features in MLP for predicting MBC subtypes and FA. The map could present the importance of each feature. The longer the yellow bar represented by this feature, the greater its weight. According to the map, the top 5 features were age, size, margin, posterior features, and echo rim. MLP, multilayer perceptron.

The AUCs of MLP for predicting pMBC, mMBC, and FA were calculated (AUCs, 0.875, 0.767, and 0.927), and the ROCs of MLP are plotted in **Figure 3**. The importance of the features is plotted in **Figure 4**.

### Clinical Use

The two models of MLP can be saved in the XML format for analysis of new data (data supplement), and the illustrations of their application are shown in data supplement (**Appendix 2**).

## Discussion

In our study, we analyzed the differences between MBC and FA, and the pairwise comparison of MBC subtypes and FA. For differentiating MBC and FA, our study observed that the sensitivity, specificity, and Youden index of age were highest, and the other eight variables exhibited modest values. Subsequently, we used the MLP to predict MBC and its subtypes and FA. Our study showed that the MLP models based on ultrasonic characteristics and age can well predict MBC and its subtypes and FA.

Our study is distinct from previous studies. Previous studies focused on reporting the correlation between ultrasonic imaging features and histological signs (12, 13). Additionally, one study proposed automated breast volume scanning and ultrasound elastography as means of predicting breast cancer, but MBC was just one of the several subtypes of breast cancer that had to be studied (20). Obviously, these studies did not investigate the differences between MBC subtypes and FA in sufficient depth.

Our study found that age and ultrasonic features, except for posterior feature and calcification, could differentiate MBC and FA based on AUCs, but the effectiveness of the ultrasonic features was moderate or poor. Obviously, the above AUCs for predicting MBC were not applicable to predict each subtype and FA. The multiple comparisons among pMBC, mMBC, and FA pointed out that there were differences in 10 variables (**Table 1**), but there was no feature that can predict MBC subtypes and FA. Therefore, single feature could not predict MBC and its subtypes and FA well. We need a more efficient tool to accomplish this task.

Before using MLP, we tried to use multinomial regression analysis, a traditional statistical method used in a similar study (21). However, the results were not satisfactory. The pseudo R^2^ of Cox and Snell was 0.495, and the p-value of Pearson test for goodness-of-fit was 0.000. The closer the R^2^ and p-value to 1, the better the fit of the model, which indicated that the fit of our model was poor and the model was meaningless.

Our study showed that the combination of ultrasonic characteristics with age by MLP can predict MBC and its subtype and FA well using MLP. Then, the two MLP maps of importance demonstrated that the importance of features was different. The top 5 features were age, size, margin, posterior features, and echo rim (**Figures 2** and **4** **)**. As far as we know, there is no study assessing the importance of ultrasonic features for MBC and its subtypes.

Age and tumor size were the strongest predictor of MBC and its subtype and FA. The older the patients are, the more likely the patients are to develop breast cancer (22, 23). Tumor size remains the important risk factor for predicting MBC, especially for pMBC. According to the biological behavior of the tumor, the more rapidly that tumor size increases, the greater the likelihood of malignancy. The size of benign tumor can remain stable for many years or increase slowly. Not circumstanced margin and calcification within masses were more positively correlated with mMBC, which is mixed with less mucin content and more no-special-type content. According to **Table 2**, the AUC of posterior features was lower than 0.5, and it cannot differentiate MBC from FA alone. However, posterior feature was one of the top 5 features in MLP. Enhanced posterior feature was the most common in pMBC because pMBC contains more extracellular mucin and has a better sound transmission ability than mMBC and FA. The presence of enhanced echogenic rim is more common in pMBC and less common in FA. In previous studies, the perifocal hyperechoic zone was associated with malignancy due to histological lymphatic invasion of the surrounding breast tissue (24, 25).

In our study, although a single feature could not predict MBC well, a strong predictive ability can be obtained by combining all features through MLP, especially in predicting FA and MBC (AUC, 0.919). Therefore, MLP was identified to be a fine classifier for the complex issue, like the previous study (15).

Our study has several limitations. First, our study’s sample size was relatively small; prospective studies with large datasets are indispensable to validate our study’s result. Second, the features did not contain clinical risk factors due to the incomplete nature of retrospective study data. Prospective studies necessitating complete datasets (BMI, serological examination) should be conducted. Third, our feature estimation was highly dependent on a subjective analysis with inevitable bias. Objective parameters’ studies need to be conducted (ultrasonic radiomics, contrast enhancement). Finally, the MLP can solve the complex classification and has the strong practicality, but the interpretability of each feature is poor. We can try other machine learnings to deal with this classification in future.

In summary, ultrasound characteristics of MBC, particularly pMBC, tend to be similar with FA. Our study found that combination of ultrasound characteristics and age by MLP can predict MBC and its subtypes and FA well. It may provide a critical insight into MBC preoperative clinical management.

## Data Availability Statement

The raw data supporting the conclusions of this article will be made available by the authors, without undue reservation.

## Ethics Statement

The studies involving human participants were reviewed and approved by the Guangdong Provincial People’s Hospital. Written informed consent to participate in this study was provided by the participants’ legal guardian/next of kin.

## Author Contributions

TL, JS, and CH conceived and designed the study. TL, SZ, and SC collected the clinical and image data. SZ, SC, and JL read and kept the record of all images. TL and JS wrote the manuscript. SP and SS reviewed and re-edited the manuscript. All authors contributed to the article and approved the submitted version.

## Funding

This work was supported by the Guangzhou Municipal Science and Technology Planning Project (CN) (202002030235) and Guangdong Medical Science and Technology Research Fund (C2018001, A2019080).

## Conflict of Interest

The authors declare that the research was conducted in the absence of any commercial or financial relationships that could be construed as a potential conflict of interest.

## Publisher’s Note

All claims expressed in this article are solely those of the authors and do not necessarily represent those of their affiliated organizations, or those of the publisher, the editors and the reviewers. Any product that may be evaluated in this article, or claim that may be made by its manufacturer, is not guaranteed or endorsed by the publisher.

## Abbreviations

MBC, mucinous breast cancer; pMBC, pure mucinous breast cancer; mMBC, mixed mucinous breast cancer; FA, fibroadenoma; MRI, magnetic resonance imaging; US, ultrasound; ROC, receiver operating characteristic curve; AUC, area under curve; MLP, multilayer perceptron.
