# Supplementary material for: Using Ultrasound-Based Multilayer Perceptron to Differentiate Early Breast Mucinous Cancer and its Subtypes From Fibroadenoma
Source: Front Oncol. 2021 Dec 1;11:724656. doi: 10.3389/fonc.2021.724656 (PMC8671140; doi:10.3389/fonc.2021.724656)
Supplement: Supplementary file 2 [file DataSheet_2.docx]

**Appendix 1.**

**The details of 10 imaging features were showed as follows.**

**Shape**

1. ***Oval*** A mass that is elliptical or egg-shaped (may include two or three undulations, i.e. gently lobulated or macro-lobulated).
2. ***Round*** A round mass is one that is spherical, ball-shaped, circular, or globular. It has an anteroposterior diameter equal to its transverse diameter; to qualify as a round mass, it must be circular in perpendicular projections.
3. ***Irregular*** The lesion shape is neither round nor oval.

**Orientation**

1. ***Parallel*** The long axis of the mass parallels the skin line. Masses that are only slightly obliquely oriented might be considered parallel.
2. ***Not Parallel*** The long axis of the mass is not parallel to the skin line. The anterior-posterior or vertical dimension is greater than the transverse or horizontal dimension. These masses can also be obliquely oriented to the skin line. Round masses are not parallel in their orientation.

**Margin**

1. ***Circumscribed*** A circumscribed margin is one that is well defined, with an abrupt transition between the lesion and the surrounding tissue. For a mass to be described as circumscribed at US, its entire margin must be sharply defined. Most circumscribed lesions have round or oval shapes.
2. ***Not Circumscribed*** If any portion of the margin is not circumscribed, the mass should be characterized as not circumscribed. A mass that is not circumscribed may further be described as having indistinct, angular, micro-lobulated, or spiculate margins, or any combination of these.

**Echogenic pattern**

1. ***Hypoechoic*** The term “hypoechoic” is defined relative to subcutaneous fat; hypoechoic masses, less echogenic than fat, are characterized by low-level echoes throughout.
2. ***Isoechoic*** Iso-echogenicity is defined as having the same echogenicity as subcutaneous fat.
3. ***Hyperechoic*** Hyper-echogenicity is defined as having increased echogenicity relative to fat or equal to fibro-glandular tissue.
4. ***Complex Cystic and Solid*** A complex mass contains both anechoic (cystic or fluid) and echogenic (solid) components.
5. ***Heterogeneous*** A mixture of echogenic patterns within a solid mass.

**Posterior Features**

1. **No Posterior Features** No shadowing or enhancement is present deep to the mass; the echogenicity of the area immediately behind the mass is not different from that of the adjacent tissue at the same depth.
2. ***Enhancement Sound transmission*** is unimpeded in its passage through the mass. Enhancement appears as a column that is more echogenic (whiter) deep to the mass.
3. ***Shadowing*** It is attenuation of the acoustic transmission. Sonographically, the area posterior to the mass appears darker.
4. ***Combined Pattern*** Some lesions have more than one pattern of posterior attenuation.

**Calcifications**

1. ***Calcifications in a mass***
2. ***Calcifications outside of a mass*** Calcifications situated in fat or fibro-glandular tissue.
3. ***Intraductal Calcifications***
4. ***None calcification***

**Echoic rim**

1. ***No echo rim*** No increased and decreased echogenicity surrounds the masses.
2. ***Enhanced echo rim*** Enhanced echogenicity surrounds the masses.

**Vascularity distribution**

1. ***Absent*** Cysts are the most common avascular lesions. Some solid masses also have little or no vascularity.
2. ***Vessels in Rim*** The blood vessels may be marginal, forming part or all of a rim around a mass.
3. ***Internal Vascularity*** Blood vessels are present within the mass. Vessels may penetrate the margin of the mass, or display an orderly or disorderly pattern within the mass.

**Vascularity grade**

1. **gradeⅠ** non-vascularity;
2. **gradeⅡ** less than 1 mm in diameter;
3. **gradeⅢ** a main vessel was seen in the area and/or several small vessels were visualized;
4. **grade Ⅳ** 4 or more vessels were visualized.

**Tumor size**

The largest diameter is merely chosen as the feature. The largest measurement should represent the longest axis of a lesion.

**Appendix 2**

After using the Multilayer Perceptron in SPSS Statistics for MLP analysis, the system can automatically generate a program in XML format. If there is new data to be analyze, you can call this application in SPSS. Our study was only a preliminary study, and its results have not yet reached the standard of clinical application. In the following, we only take the program with the file name as clinical use (MBC subtype) as an example to illustrate the related usage of such programs:

The first step: open new data with SPSS Statistics→Utilities→Scoring Wizard.


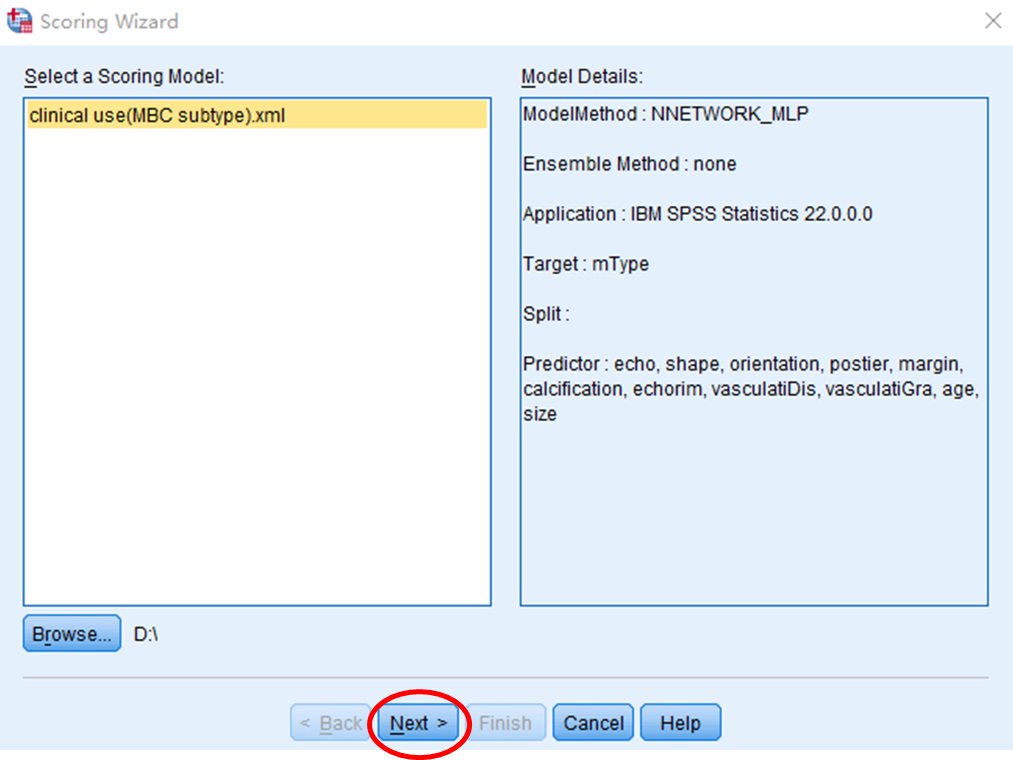


The second step: found and select the XML format file, click the “Next”.


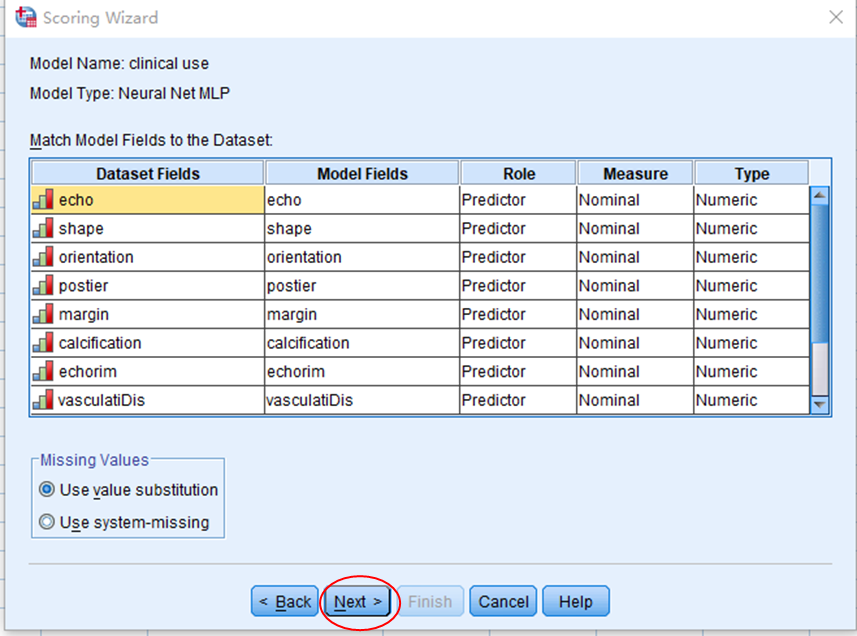


The third step: check whether the definitions of variables in new data are accurate, click the “Next”.


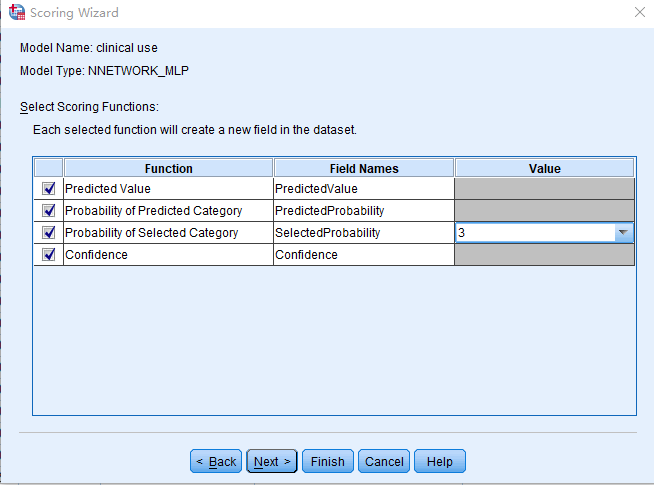


The fourth step: select the appropriate options as needed. The values of SelectedProbability are 1, 2 and 3, which represent FA, pMBC and mMBC, respectively.

**Predicted value (PredictedValue)**. The predicted value of the target outcome of interest.

**Probability of predicted value (PredictedProbability).** The probability of the predicted value being the correct value.

**Probability of selected value (SelectedProbability).** The probability of the selected value being the correct value.

**Confidence.** A probability measure associated with the predicted value of a categorical target.


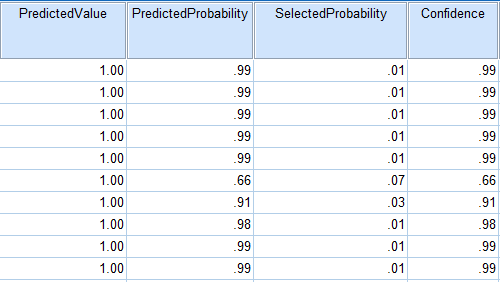


Finally, the corresponding result columns are added to the new data.
